# mTORC1-Inhibition Potentiating Metabolic Block by Tyrosine Kinase Inhibitor Ponatinib in Multiple Myeloma

**DOI:** 10.3390/cancers14112766

**Published:** 2022-06-02

**Authors:** Uddin Md. Nazim, Kausik Bishayee, Jieun Kang, Dongkwan Yoo, Sung-Oh Huh, Ali Sadra

**Affiliations:** Department of Pharmacology, College of Medicine and Institute of Natural Medicine, Hallym University, Chuncheon 24252, Korea; nazim@hallym.ac.kr (U.M.N.); kausik@hallym.ac.kr (K.B.); 40442@hallym.ac.kr (J.K.); ydg9611@naver.com (D.Y.)

**Keywords:** glycolysis, multiple myeloma, ponatinib, sirolimus, mTORC1

## Abstract

**Simple Summary:**

From a screen for metabolic inhibition by a panel of approved anticancer drugs and combining the lead compound with a mammalian target of rapamycin complex 1 (mTORC1) inhibitor, we demonstrated that the combination of ponatinib and sirolimus leads to synergistic tumor growth inhibition in a mouse xenograft tumor model of multiple myeloma. The rationale of combining the two drugs was to prevent metabolic escape due to glycolysis reprogramming and residual oxidative phosphorylation (OXPHOS). The robust increases in reactive oxygen species (ROS) due to a block in glycolysis were shown to be the lead contributor of cell viability loss. The drug combination in the doses used displayed no overt toxicity in the treated animals.

**Abstract:**

Studies in targeting metabolism in cancer cells have shown the flexibility of cells in reprogramming their pathways away from a given metabolic block. Such behavior prompts a combination drug approach in targeting cancer metabolism, as a single compound may not address the tumor intractability. Overall, mammalian target of rapamycin complex 1 (mTORC1) signaling has been implicated as enabling metabolic escape in the case of a glycolysis block. From a library of compounds, the tyrosine kinase inhibitor ponatinib was screened to provide optimal reduction in metabolic activity in the production of adenosine triphosphate (ATP), pyruvate, and lactate for multiple myeloma cells; however, these cells displayed increasing levels of oxidative phosphorylation (OXPHOS), enabling them to continue generating ATP, although at a slower pace. The combination of ponatinib with the mTORC1 inhibitor, sirolimus, blocked OXPHOS; an effect also manifested in activity reductions for hexokinase 2 (HK2) and glucose-6-phosphate isomerase (GPI) glycolysis enzymes. There were also remarkably higher levels of reactive oxygen species (ROS) produced in mouse xenografts, on par with increased glycolytic block. The combination of ponatinib and sirolimus resulted in synergistic inhibition of tumor xenografts with no overt toxicity in treated mice for kidney and liver function or maintaining weight.

## 1. Introduction

The metabolic characteristic of cancer cells compared to normal, differentiated cells impinges on the preference of cancer cells to use glycolysis even in the presence of adequate supplies of oxygen, rather than using oxidative phosphorylation in mitochondria to produce adenosine triphosphate (ATP) [1]. This characteristics of cancer cell is called the Warburg effect, and it is controlled by multiple oncogenes [2]; it is the resultant upregulation of a number of rate-limiting enzymes in the glycolysis pathway [3]. Additional manifestations of the Warburg effect include relatively high levels of glucose uptake and increased secretion of lactate by the cancer cell. To enable this, the cancer cells accumulate plasma membrane mono-carboxylate transporters (MCTs) that enable lactate export from the cell and allow the cell to function without becoming acidotic [4]. Additional cancer cell metabolic adaptations include altered metabolism and uptake of certain amino acids [5]. From these observations, targeting the upregulated glucose uptake and glycolysis in cancer cells is thought of as adding a measure of selectivity to cancer therapy, while leaving the normal cells largely unaffected [6].

Multiple myeloma (MM) is an incurable cancer of fast-dividing plasma cells present in the bone marrow, and it frequently develops resistance to various approved drugs, with almost all patients experiencing relapse and succumbing to the disease [7]. In terms of numbers, MM is the second most common blood-borne malignancy, affecting millions globally [8]. Although chemotherapies, proteasome inhibitors, and immunotherapies have meaningfully improved the 5-year survival in MM patients, as noted above, MM uniformly proves fatal; and new therapeutic drug regimens are required to further increase survival from this cancer [7]. There is evidence that the Warburg effect is operational in MM, as is also seen with the upregulation of glucose uptake and certain glycolysis enzymes [9,10].

To test the effects of currently approved drugs on the metabolic characteristic of multiple myeloma cells, we performed a metabolic screen of a library of approved anti-cancer compounds on these cells (Approved Oncology Drugs Set; dtp.cancer.gov accessed on 7 December 2019; National Cancer Institute (NCI), Bethesda, MD, USA; plate key 4891047). From what we know, developing resistance to drugs is a hallmark of MM, and we chose a combination drug treatment by coupling the outcome of the NCI compound screen with another compound, namely sirolimus, a specific inhibitor of mammalian target of rapamycin complex 1 (mTORC1). We elected to focus on mTORC1, as it is the signaling gatekeeper for the upstream phosphatidylinositol 3-kinase (PI3K)/ a serine/threonine protein kinase (AKT) and mitogen-activated protein kinase (MAPK) growth pathways [11], and these regulate the expression of a number of glycolytic enzymes for the enhancement of glycolysis and promotion of tumor cell growth [12]. Moreover, mTORC1 has also been implicated in allowing cells to escape glycolysis block by various pharmaceutical means [12]. In multiple myeloma, mTOR knockdown reduces the proliferation of MM cells and inhibits the rate of glycolysis [13].

Several mTOR inhibitors have been approved by the United States Food and Drug Administration (US FDA) for a number of cancer types [14,15]; however, as single agents, these compounds have shown only limited efficacy in clinical trials, only slowing the growth of the tumors rather than being cytotoxic [16]. From our library of compounds, ponatinib uniformly blocked glycolysis and displayed synergistic efficacy when combined with the mTORC1 inhibitor, sirolimus, in treated MM cells and in the mouse MM xenograft. This combination displayed effective downmodulation of key rate-limiting glycolytic enzymes, notably hexokinase 2 (HK2) and glucose-6-phosphate isomerase (GPI). From what we know of ponatinib, it is a multi-targeted kinase inhibitor, originally developed for inhibiting mutant forms of breakpoint cluster region gene (BCR)-Abelson protooncogene (Abl) fusion kinase [16,17]; it has also been shown to block Abl, fibroblast growth factor receptor (FGFR), and a number of other tyrosine kinases [18,19,20].

In our study, the increased levels of blocked glycolysis were commensurate with vigorous increases in reactive oxygen species (ROS) levels, which we showed to have sizably reduced cell viability, and demonstrated recovery from viability loss when the cells were in presence of ROS scavenger, N-acetyl-l-cysteine (NAC). Finally, the combination therapy of ponatinib with sirolimus in multiple myeloma displayed synergistic inhibition of xenograft tumor growth and displayed no overt signs of toxicity in treated mice in terms of weight loss or for parameters of liver or kidney function. Our study points to the promise of a combination therapy of two compounds obtained from a metabolic screen in treatment of MM, targeting the noted parameters of increased glycolysis in these cells, synergizing to have impressive blocks in tumor growth, and having no detected systemic toxicity. In this regimen of therapy, we believe that a personalized approach in dosing would be required. Fortunately, these two compounds are well studied as approved anticancer drugs, and there are a host of analytical assays available to measure their bio-distribution and confirm their target inhibition from serum samples.

## 2. Materials and Methods

### 2.1. Ethics Approval and Consent to Participate

The animal studies were conducted according to the approved protocols by the Institutional Animal Care and Use Committee (IACUC) of Hallym University (Chuncheon, Korea) (protocol approval number: Hallym2020-55).

### 2.2. Gene Signature and Survival Probability for Multiple Myeloma

*HK2* and *GPI* gene expression profile and overall survival in multiple myeloma patients were from R2 database (http://r2.amc.nl; Department of Oncogenomics, Academic Medical Center, accessed on 12 December 2021) (Table S1). Correlation analysis for expression was with Prism 5 software (GraphPad, San Diego, CA, USA).

### 2.3. Cell Culture

Human MM (RPMI8226, U266) and HEK293 cell lines were obtained from Korea cell line bank (KCLB) and the American Type Culture Collection (ATCC) (Manassas, VA, USA). Cells were cultured in RPMI-1640, DMEM (Gibco BRL, Grand Island, NY, USA) medium supplemented with 10% heat-inactivated fetal bovine serum (FBS) (Gibco BRL), and antibiotics (100 μg/mL penicillin–streptomycin) (Gibco BRL). Cells were grown and maintained at 37 °C, in a humidified atmosphere of 95% air and 5% CO_2_.

### 2.4. NCI Drug Plate Screening

Compounds from Approved Oncology Drugs Set (dtp.cancer.gov accessed on 7 December 2019; NCI plate key 4891047) were for screening and donated by NCI under their Development Therapeutics Program (DTP) (National Cancer Institute, Bethesda, MD, USA). RPMI8226, U266, or HEK293 cells were seeded in 96-well plates, at 1 × 10^4^ cells/well, in culture medium, with drug treatment for 72 h. The metabolic activity of the cells was measured by using Quanti-Max assay kit (Biomax, Seoul, Korea) with fluorescent intensity read from Ext-450 nm. All the experiments were performed 2 or more times to assay reproducibility.

### 2.5. RNA Extraction and qPCR

Total mRNA was isolated by using the miRNeasy Mini Kit (Qiagen, Germantown, MD, USA), followed by cDNA synthesis, using the miScript II RT Kit (Qiagen), according to the manufacturer’s protocol. Levels of target mRNA were evaluated with AccuPower GreenStar qPCR kit (Bioneer, Daejeon, Korea). β-actin (ACTB) was used as the internal control. All the experiments were performed 2 or more times to assay reproducibility. Primers in qPCR are listed in Table S2.

### 2.6. qPCR Screening Array for Glucose Metabolism

RPMI8226 cells were treated with either control or ponatinib (1 µM) for 12 h, total RNA was extracted, and cDNA was prepared. The glucose metabolism gene set expression changes for 92 genes (AccuTarget qPCR Screening Kit for Glucose Metabolism, SH-0000-10, Bioneer) was evaluated by qPCR, using the AccuPower Green Star qPCR kit. Plate map for glucose metabolism gene set for qPCR is in Table S3.

### 2.7. Measurement of ROS Levels

Cellular ROS levels were quantified by 2′,7′-dichlorofuorescein diacetate (DCFDA) (Sigma-Aldrich, St. Louis, MO, USA). The cells were seeded at 20,000 in 96-well plate format and in replicates. They were then incubated with various drugs for the given times. The ROS scavenger treatment with N-acetyl-l-cysteine (NAC) (Sigma) was at 3 mM, with the cells being pretreated for 1 h before the DCFDA and viability reads. Hydrogen peroxide (H_2_O_2_) treatment at 50 μM for 3 h, being an ROS inducer, served as a positive control for the ROS assays. To measure the ROS levels, to each well, 25 μM DCFDA was added and incubated for 30 min. Plate well fluorescence was measured at 488 nm/525 nm excitation/emission in a microplate reader. The values from the well replicates were plotted for their mean ± SEM.

### 2.8. Metabolically Active/Dead Cell Staining

RPMI8226 and U266 cells were seeded in 96-well plates, at 1 × 10^4^ cells/well, in culture medium, and fluorescent stains were used to stain the RPMI8226 and U266 cells to determine cell health and viability: calcein-AM (Life Technologies, Carlsbad, CA, USA) for metabolically active cells, and propidium iodide (PI) for dead cells (Life Technologies). The cells were stained for 30 min in the dark at 37 °C. Images were then captured for green (metabolically active cells) and red (dead cells), using a fluorescence microscope. Image analysis was performed with ImageJ software (National Institutes of Health, Bethesda, MD, USA). All the experiments performed 6 or more times to confirm reproducibility.

### 2.9. Glycolysis Biochemical Assays

Glycolysis markers of glucose uptake, pyruvate, lactate production, and ATP levels were performed according to the manufacturer’s protocol for each assay. HK2 and GPI activity assays were performed on RPMI8226 and U266 cells according to the manufacturer’s instructions. All the experiments were performed 2 or more times to assay reproducibility. Glycolysis markers applied for assays are listed in Table S4.

### 2.10. Western Blots

MM cells were seeded in 6-well plates, at a density of 1 × 10^6^ cells per well. Cells were washed with PBS on ice, their cell pellets were lysed in RIPA buffer (iNtRON Biotechnology, Seongnam, Korea) in the presence of protease and phosphatase inhibitors (5872S, Cell Signaling Technology, Danvers, MA, USA), and the lysate supernatants were kept after centrifugation at 4 °C. From the protein lysates, a conventional Western blot experiment involving SDS–PAGE, electro-transfer onto a PVDF membrane; then membrane blocking/antibody probing was performed, and the protein bands were visualized by enhanced chemiluminescence (ECL) (Luminata Forte, Millipore, Burlington, MA, USA) and detection with FUSION FX-Western Blot imaging system (Vilber, Collégien, France). β-actin (ACTB) was used as the internal control. The antibodies used for Western blotting are listed in Table S5.

### 2.11. Immunohistochemistry

After sectioning tissues, they were fixed in 4% paraformaldehyde (Sigma) and then incubated in 15% and 30% sucrose (Sigma), each at 4 °C overnight. The tissues were then mounted in OCT blocks, followed by sectioning at 5 μm thickness with a cryo-sectioning machine. Antigen retrieval was then performed with 0.05% trypsin (Sigma) for 15 min at 37 °C. The samples were then blocked in 10% goat serum (Jackson ImmunoResearch, West Grove, PA, USA) for 1 h. Incubations with primary antibodies were performed overnight at 4 °C. The slides were then incubated with DAPI nuclear stain at 1 µg/mL and Alexa Fluor-conjugated secondary antibodies (Invitrogen, Thermo Fisher, Waltham, MA, USA) for 3 h at room temperature and were mounted. The images were obtained by a confocal microscope (LSM 710-Zeiss) (Zeiss, Ostfildern, Germany), with picture capture, using the ZEN 2.6 software (Zeiss). Image analysis was performed via ImageJ (NIH). Antibodies applied for Immunohistochemistry are listed in Table S5.

### 2.12. Hematoxylin–Eosin Staining

After sectioning, the tissues were fixed in 4% paraformaldehyde and were then dehydrated. After the overnight incubation, the samples were mounted in OCT blocks sectioned at 5 μm thickness, using a cryo-sectioning machine. The slides were placed in alcohol to distilled water gradient. Finally, histological analysis by using a hematoxylin-and-eosin staining kit (ab245880) (Abcam, Cambridge, UK) was performed.

### 2.13. Subcutaneous Xenograft Model

All animal study protocols were reviewed and approval by the Institutional Animal Care and Use Committee (IACUC) of Hallym University (Chuncheon, Korea) (protocol approval number Hallym2020-55). Five-week-old female BALB/C nude mice were obtained from DBL Korea (Eumseong, Korea). The mice were kept in specific-pathogen-free (SPF) conditions in spacious cages, with free access to food and water and 12 h day/night cycle, with controlled optimal temperature/humidity maintained in their rooms. For tumor implantation, human multiple myeloma RPMI8226 cells were cultured, harvested, and resuspended in 20% Matrigel (3433-001-R1, Trevigen, Gaithersburg, MD, USA) in PBS and were subcutaneously injected at 5 × 10^6^ cells into the flank region of the nude mice. Treatment with the control or the compounds started when the tumors were palpable. The mice were also randomly divided into four groups. Drug treatments were with intraperitoneal injection of 100 μL PBS or ponatinib (10 mg/kg) and/or sirolimus (5 mg/kg) for three times per week. Tumor volumes were measured at the indicated time intervals, with the tumor volumes calculated as follows: V(volume) = (length × width2)/2. At the point of sacrifice, mouse body weights were also recorded. For the blood toxicity tests, the mice were tested with intraperitoneal injection with 100 μL PBS (Gibco BRL) or ponatinib (Biosynth Carbosynth, Staad, Switzerland) (10 mg/kg) and sirolimus (Alfa Aesar, Thermo Fisher, Ward Hill, MA, USA) (5 mg/kg), three times a week and for two weeks. Liver and kidney function was evaluated with serum levels of physiochemical indices, including blood alanine aminotransferase (ALT), aspartate aminotransferase (AST), blood urea nitrogen (BUN), and creatinine (Cr) levels measured analytically by a commercial vendor (T&Pbio, Gwangju-si, Korea).

### 2.14. Statistical Analysis

Statistical analyses were performed by using GraphPad Prism version 5 software (GraphPad, La Jolla, CA, USA). The analytical data are expressed as mean ± standard error, compared statistically by Student’s *t*-test (2 groups) and ANOVA with “Tukey’s Multiple Comparison Test” (more than two groups). For normality of distribution, the program performed a Shapiro-Wilk test. A post hoc achieved power analysis was performed with G*Power program version 3.1.9.4 (Franz Faul, University of Kiel, Germany). Significant differences are indicated for *p* < 0.05.

## 3. Results

### 3.1. NCI-Library Drug Screening Was Performed in Reducing Metabolism

**Figure 1:**
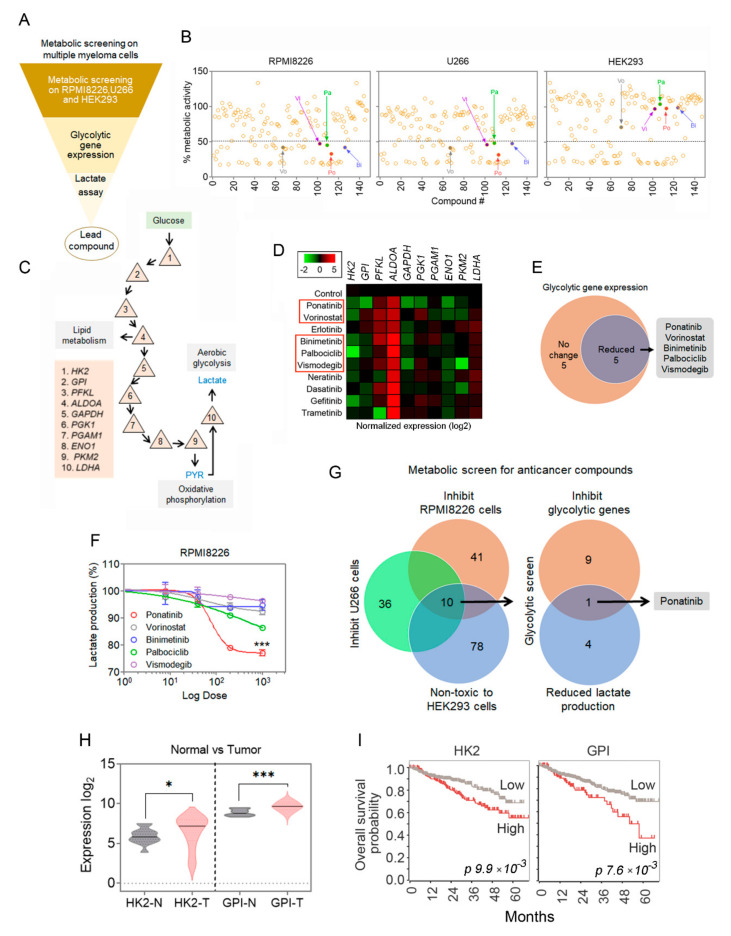
Metabolic screen of multiple myeloma cells. (**A**) Schematic for metabolic screening targeting glycolysis pathway in MM cells. (**B**) Assay for metabolic efficiency of compounds from NCI-drug library on RPMI8226 and U266 MM cells; HEK293 cells were used to determine the toxicity of the compounds. Each point represents a single compound from the library; the assay was performed in duplicate for reproducibility (Vo = vorinostat, Vi = vismodegib, Pa = palbociclib, Po = ponatinib, Bi = binimetinib). Cells were incubated with the drug compounds for 72 h for the assay. (**C**) Schematic diagram of the glycolysis pathway for the enzymatic links. (**D**,**E**) The top ten selected drug candidates were screened for inhibition of glycolytic genes by qPCR. The drug treatment was for 12 h on RPMI8226 cells. (**F**) The top 5 candidates were further screened for inhibition in lactate production using a lactate assay kit on RPMI8226 cells; drug treatment was for 24 h. Data are presented as mean ± SEM; *t*-test: *** *p* < 0.001. (**G**) Venn diagram shows ponatinib was the best candidate compound for glycolysis gene and lactate production inhibition. (**H**) HK2 and GPI signals were analyzed in normal and MM cells in cancer patients. The patient data were obtained from the R2 platform (hgserver1.amc.nl) (GEO ID: GSE2658). N = normal tissue; T = tumor. Data are plotted as mean with the shaded distributions; * *p* < 0.05, *** *p* < 0.001, provided by R2 platform search engine (via one-way ANOVA on ranks). (**I**) Kaplan–Meier overall survival plots of MM patients with respect to HK2 and GPI RNA message levels are shown. Significance p-values are indicated in the figure, provided by R2 platform search engine (hgserver1.amc.nl) (GEO ID: GSE2658) (via one-way ANOVA on ranks).

In this study, we aimed to screen the panel of approved anticancer drugs that potentially inhibit glycolysis in multiple myeloma cells. To find the glycolytic modulating drugs, we performed a metabolic screen in two MM cell lines, RPMI8226 and U266, and the non-tumor HEK293 cells. Furthermore, modulation of glycolysis was confirmed with various assays and gene-expression profile changes; the details of the protocol are described in the figure legend (Figure 1A). We screened a compound library from NCI for modulation of metabolic activity of the treated cells and chose the 72 h post-treatment for assay of the metabolic activity of 10,000 cells. All the compounds were tested at 1 µM and were scored as hits if they inhibited the cell metabolic activity relative to the control by 50% or more. These compounds represent diverse drug classes, including antineoplastic drugs, antibiotics, inhibitors of histone deacetylases (HDACs), and kinase and DNA topoisomerase inhibitors. The primary screening (Figure 1B) and validation of top hits were then performed. Overall, 41 compounds were seen to reduce the metabolic activity of RPMI8226 cells, 36 compounds did the same for U266 cells, and 78 compounds that did not affect the metabolic activity of HEK293 cells at the 1 µM and 72 h conditions used for the assay. From these subsets, there were 10 compounds that were in common. For these 10 compounds, 5 were seen to suppress expression of a number of glycolytic pathway genes for the test of 10 genes via PCR (Figure 1C–E). The glycolytic enzymes are sequentially listed in the accompanying schematic (Figure 1C). From these five compounds, in a dose dependent assay for lactate production in the RPMI8226 cells, ponatinib showed the most potency and was thus selected as our lead compound for further analysis (Figure 1F,G). From a search of MM patient databases, increased levels of glycolysis were presumed with augmented levels of HK2 and GPI RNA message in tumor versus normal tissue (Figure 1H); the overall survivability of the patient was also worse with augmented RNA message levels for HK2 and GPI (Figure 1I), implying increased levels of glycolysis with respect to these two enzymes having a negative prognosis on survivability.

### 3.2. Ponatinib Regulates Glycolysis and Related Gene Expressions

**Figure 2:**
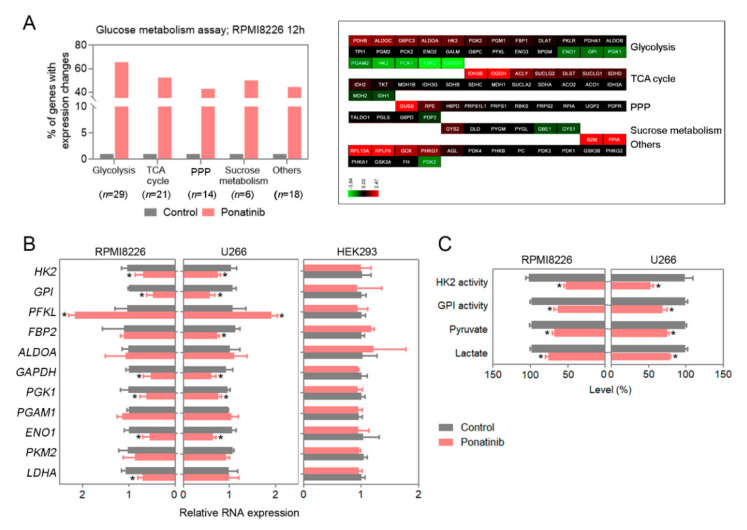
Ponatinib inhibits the glycolysis pathway in multiple-myeloma cells. (**A**) Glucose metabolism array was performed on RPMI8226 cells, using Bioneer gene-expression array plates. Cells were exposed to ponatinib (1 μM) for 12 h, and total mRNA was extracted. The gene expression was analyzed by using qPCR. The histogram represents the ‘% changes’ in gene expression for different glucose metabolism pathways. (**B**,**C**) Ponatinib and vehicle control, dimethylsulfoxide (DMSO), treatment were for 12 h. (**B**) Glycolytic gene-expression changes were quantified by using qPCR. (**C**) HK2 and GPI activity and pyruvate and lactate production were examined by using specific assay kits. Data are presented as mean ± SEM; *t*-test: * *p* < 0.05.

Multiple studies have revealed that the Warburg effect is in effect in many tumors, including multiple myeloma [21]. As such, we performed a metabolism pathfinder assay based on RNA changes, and the result of 92 genes in a PCR array for ponatinib treatment at 1 µM for 12 h on RPMI8226 cells is shown (Figure 2A). We noticed that a number of glycolysis-related genes are downregulated with ponatinib compared with the control. The follow-up experiment with ponatinib treatment of the cells (RPMI8226 and U266) led to significant downmodulation of key rate-limiting genes, i.e., HK2 and GPI, compared with the control cells (HEK293) (Figure 2B). For our positive control, we treated the cells with the glycolysis inhibitor 2-deoxyglucose (2DG) for 12 h, and 2DG also led to significant reductions in RNA levels for the glycolytic enzymes in RPMI8226 cells (Figure S1). Ponatinib treatment also translated to functional measures of glycolysis (levels of HK2, GPI, fructose-1,6-bisphosphatase-2 (FBP2), phosphoglycerate kinase 1 (PGK1), and enolase 1 (ENO1); activity of HK2 and GPI; and generation of pyruvate and lactate) at 24 h post-treatment compared with control in RPMI8226 and U266 cells (Figure 2B,C).

### 3.3. Ponatinib-and-Sirolimus Combination Treatment Inhibits Glucose Uptake and the Markers of Glycolysis in MM Cells

**Figure 3:**
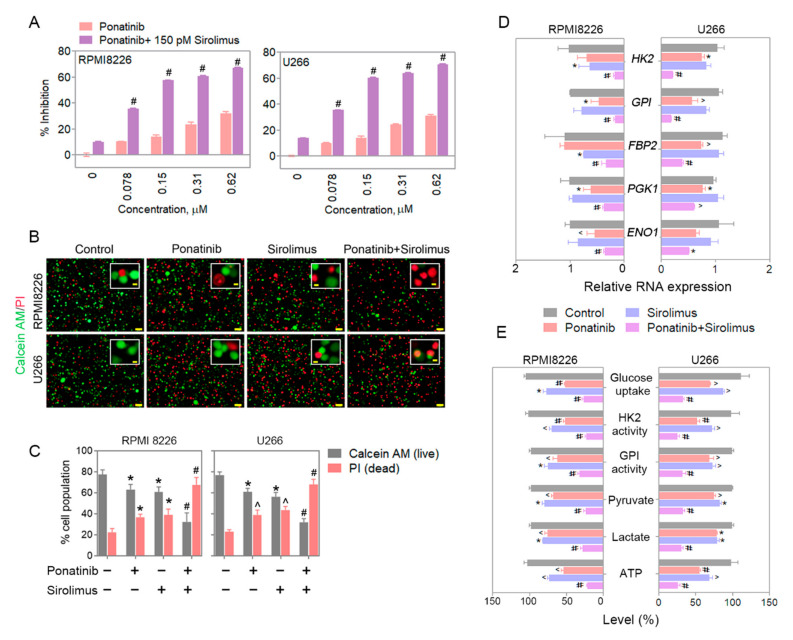
Sirolimus potentiates ponatinib-mediated glycolysis inhibition and induces multiple myeloma cell death. (**A**) Metabolic assay was performed on RPMI8226 and U266 cells in the presence of variable doses of ponatinib alone or with 150 pM sirolimus. The histograms represent the inhibition percentages; treatment was for 72 h. Sirolimus at 150 pM potentiates the ponatinib inhibition on metabolic activity significantly. (**B**) Calcein AM and PI staining show a combination of ponatinib and sirolimus significantly increases the dead cell population in the cultures of MM cells. (**C**) Quantification for Calcein AM and PI-positive cell populations. Cells were counted by using ImageJ software and plotted as histograms, using GraphPad Prism 5 software. Scale bars for the large field are 50 µm; those for the magnified inset are 5 µm. (**D**) RPMI8226 and U266 cells were treated with ponatinib and/or sirolimus for 12 h. The glycolytic gene-expression changes were examined by qPCR. (**E**) RPMI8226 and U266 cells were treated with ponatinib and/or sirolimus for 24 h. The HK2 and GPI activity was measured by activity assay kits. Glucose, ATP, lactate, and pyruvate levels were quantified by specific assay kits. Doses used for ponatinib and sirolimus were 300 nM and 150 pM, respectively. Data are presented as mean ± SEM; *t*-test: * *p* < 0.05, ^ *p* < 0.01, and # *p* < 0.001.

As mentioned in the Introduction, mTORC1 has been implicated in enabling cells to bypass certain blocks in glycolysis by various pharmaceutical agents [12]; thus, concomitant treatment of the MM cells with an mTORC1 inhibitor may potentiate the effects of our glycolysis block by ponatinib alone. In addition, mTOR knockdown leads to reduced proliferation of MM cells and also reduces the rate of glycolysis [13]. In the present study, with ponatinib and mTORC1 inhibitor, sirolimus, co-treatment of either RPMI8226 or U266 cells, a significant decrease was detected in the viability of the cells treated with either compound alone and in a dose-dependent fashion (Figure 3A), and there was synergy in their effect, according to their CI score (Figure S2A–C). In terms of the effects on metabolic activity, the best inhibitions were also seen by the combination of the two compounds rather than each alone in both RPMI8226 and U266 cells (Figure 3B,C). RPMI8226 and U266 cells treated with suboptimal doses of ponatinib-and-sirolimus combination also had augmented reductions in mRNA for key glycolytic enzymes when compared with single compound treatments (Figure 3D and Figure S2D). Moreover, the markers of glucose metabolism and glycolysis were similarly inhibited by several fold by the combination of the two compounds (glucose uptake; HK2 and GPI activity; and production of pyruvate, lactate, and ATP) (Figure 3E).

### 3.4. Combination Compounds Reduce Activated AKT and mTORC1 Levels, along with the Protein Levels of Glycolysis Rate-Limiting Enzymes, HK2 and GPI, in Treated MM Cells

**Figure 4:**
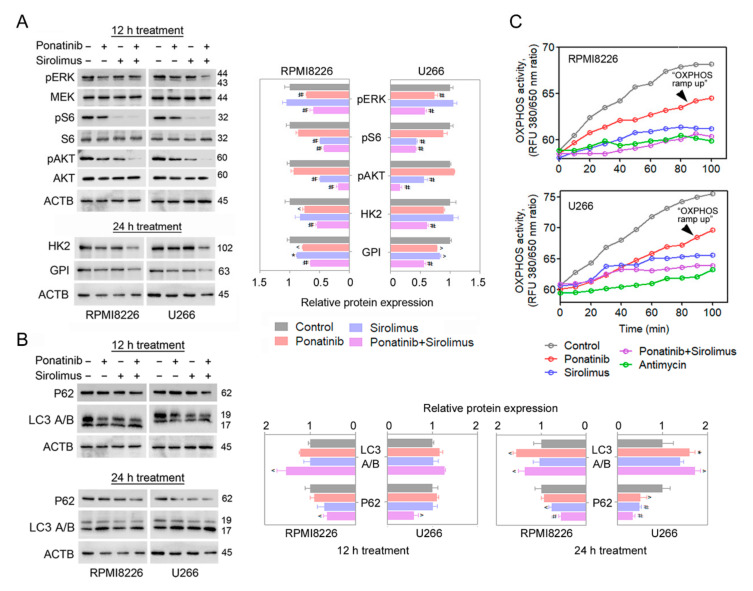
Ponatinib and sirolimus inhibit AKT/mTORC1 pathways and residual oxidative phosphorylation (OXPHOS) in multiple myeloma cells. (**A**) Ponatinib at 300 nM and/or 150 pM sirolimus was/were administrated to RPMI8226 and U266 cells for 12 or 24 h. The activity of ERK/AKT/mTORC1 pathways was quantified by specific phosphorylated antibodies with Western blot. Expression was quantified by ImageJ and represented as a histogram. HK2 and GPI expression was determined after 24 h of treatment and quantified. (**B**) RPMI8226 and U266 cells were treated with ponatinib, sirolimus, or with their combination for 12 and 24 h. Changes in LC3 A/B and P62 expression were quantified by Western blotting. Expression was quantified by using ImageJ software and plotted as histograms. (**C**) OXPHOS was measured in vitro, using a specific kit after 24 h of treatments; antimycin was used as a positive control for OXPHOS inhibition. Ponatinib and sirolimus doses were 300 nM and 150 pM, respectively. The uncropped Western blots are shown in Supplementary Figures S4 and S5. Data are presented as mean ± SEM; *t*-test: * *p* < 0.05, ^ *p* < 0.01, and # *p* < 0.001.

To uncover the upstream signaling pathways affected by ponatinib, sirolimus, and their combination, a Western blot survey of AKT, mTORC1, and MAPK/ extracellular signal-regulated kinase (ERK) pathways was performed in RPMI8226 and U266 cells. We also checked the protein-level changes for HK2 and GPI, the two glycolysis rate-limiting enzymes (Figure 4A). Changes in the markers of autophagy, P62 (encoded by gene *SQSTM*) and LC3 A/B (encoded by *MAP1LC3B* gene), were also analyzed, as activity of mTORC1 is a known modulator of autophagy, whose levels can modulate survival in affected cells [22] (Figure 4B). Treatment of RPMI8226 and U266 cells at 12 and 24 h with ponatinib and sirolimus led to significant inhibitions in levels of activated AKT (pAKT, Ser473) and mTORC1 activity marker (pS6, Ser240/244) in both cell types. The levels of the glycolytic enzymes HK2 and GPI were also significantly reduced by the combination treatment. The changes in the levels of activated ERK (pERK, Thr202/Tyr204) and those of the markers of autophagy (LC3 A/B and P62) were also reduced, but not consistently for both cell types (Figure 4A,B).

### 3.5. Combination Compounds Reduces Levels of Oxidative Phosphorylation in Treated MM Cells, Blocking the Additional Source of ATP

Oxidative phosphorylation (OXPHOS) is the additional source of ATP in cancer cells, carried out inside mitochondria [23]. Although it is not the dominant pathway for making ATP in various cancer cells, including MM [9,10], there have been reports of upregulated OXPHOS, “OXPHOS escape” in certain cancer cells undergoing a glycolysis block, by rechanneling glucose flux and providing renewed ATP production and production of biomass [12,24]. With the combination compounds, sirolimus and ponatinib, OXPHOS was similarly blocked in RPMI8226 and U266 cells, similar to the levels of the positive control, antimycin A (Figure 4C). This demonstrated that, besides a blocked glycolysis and reduced glucose uptake, an alternative possible source of ATP was also blocked in the ponatinib/sirolimus-treated cells, and a reprogramming of metabolism to “OXPHOS escape” (upregulated OXPHOS) was not seen.

### 3.6. Combination Compounds Results in Sizable Increases in ROS Levels in Treated Cells

**Figure 5:**
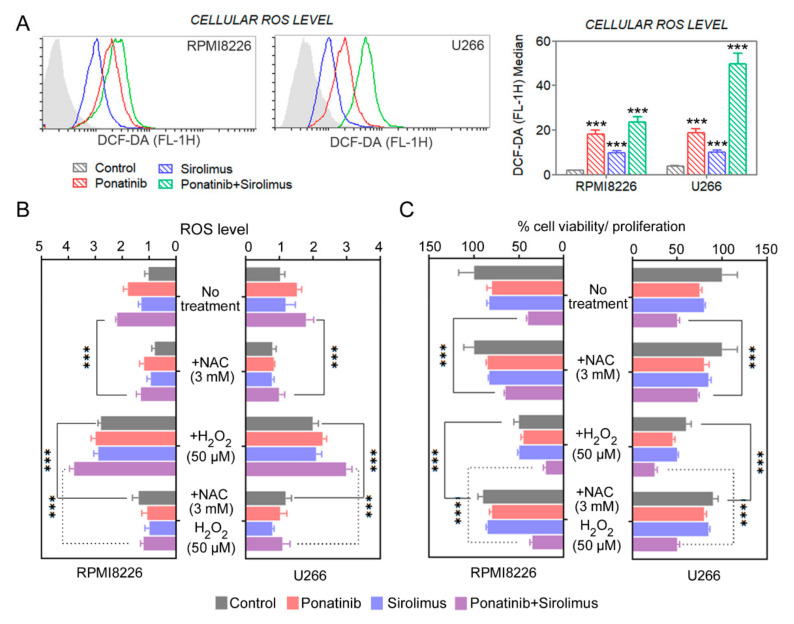
Ponatinib and sirolimus lead to accumulation of cellular ROS in multiple myeloma cells. (**A**) ROS accumulation in cells was quantified with flow cytometry after DCFDA staining. (**B**,**C**) Cellular ROS were measured by DCFDA fluorescence, with or without H_2_O_2_ (50 μM) and/or NAC (3 mM) presence, and cell viability was assayed. Data are presented as mean ± SEM; *t*-test: *** *p* < 0.001.

Furthermore, mTOR inhibitors can bring about toxic levels of oxidative stress for cell death [25]. Regarding whether reactive oxygen species (ROS) were also increased by the combination treatment of ponatinib/sirolimus, the ROS levels were measured with DCFDA fluorescence in RPMI8226 and U266 cells in the presence of ponatinib, sirolimus, or their combination (Figure 5A,B). It was noted that there was a sizable increase in levels of ROS, particularly for ponatinib and the combination of the two drugs; however, it was possible to block this increase with the addition of the ROS scavenger N-acetyl-l-cysteine (NAC) (Figure 5B). The addition of an exogenous source of oxidative stress, namely H_2_O_2_, increased the magnitude of ROS changes from the ponatinib/sirolimus combination when compared with the cells treated with a single compound alone, thus implying that the combination drugs are also best in blocking the detox mechanisms of oxidative stress (Figure 5B). Again, the addition of NAC prevented the accumulation of ROS under all conditions, even for H_2_O_2_, showing that we did not overwhelm the cells with excessive levels of H_2_O_2_ and that NAC salvage was possible (Figure 5B). We conclude that there is a significant increase in oxidative stress due to free-radical formation in ponatinib/sirolimus-treated cells. This could be due to a block in glycolysis, as glycolysis inhibition commonly augments ROS levels from reduced levels of pyruvate, acting as an antioxidant [26,27].

### 3.7. Reducing the ROS Levels with Antioxidant NAC Partially Recovers Viability Loss Due to Combination Compound Treatment in MM Cells

ROS levels were significantly increased in RPMI8226 and U266 cells treated with ponatinib combined with sirolimus as compared to single treatment (Figure 5A). To determine the contribution of oxidative stress to viability loss in the ponatinib/sirolimus-combination-treated MM cells, RPMI8226 and U266 cells were pretreated with the antioxidant NAC before exposing the cells to the two compounds (Figure 5B). NAC prevented some of the viability loss due to ponatinib/sirolimus co-treatment, but not completely. In the presence of additional levels of ROS from exogenous H_2_O_2_, the cytotoxic effect of ponatinib and sirolimus was significantly amplified, suggesting that the ROS detox mechanisms were overwhelmed by the sirolimus/ponatinib combination (Figure 5C), triggering a catastrophic oxidative stress and subsequent cytotoxicity.

### 3.8. Synergistic Tumor Growth Inhibition Is Seen for Ponatinib-and-Sirolimus Combination Treatment for RPMI8226 Xenografts in Mice

**Figure 6:**
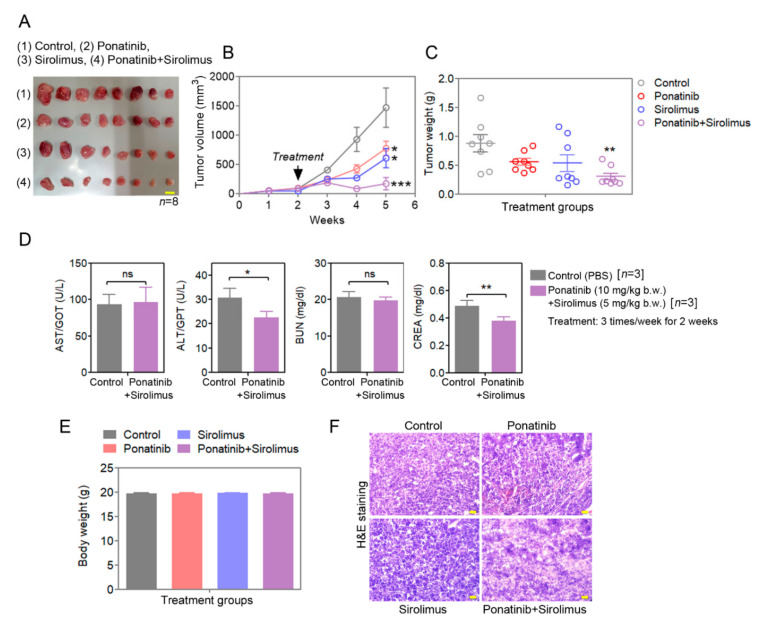
Ponatinib-and-sirolimus combination effectively reduces the multiple-myeloma tumor burden in athymic nude mouse. (**A**) RPMI8226 tumor images for different treatment groups. Scale bar corresponds to 1 cm (picture of *n* = 8 shown; there were 10 mice per arm). (**B**) Tumor volume was measured at different time points during treatments. The tumor volume was calculated and plotted as a curve, using GraphPad Prism 5 software. (**C**) The terminal tumor weight was measured and plotted by using GraphPad Prism 5 software. Achieved power analysis using G*Power 3.1.9.4 is shown in Table S6. (**D**,**E**) Different toxicity tests were performed for the ponatinib-and-sirolimus combination treatment group. Aspartate aminotransferase (AST), alanine aminotransferase (ALT), blood urea nitrogen (BUN), and creatinine (CREA) levels in the serum were measured biochemically. Terminal mouse body weight was measured. Data are presented as mean ± SEM; *t*-test: * *p* < 0.05, ** *p* < 0.01, and *** *p* < 0.001. ns: not significant. (**F**) H&E staining was performed on fixed tumor tissue samples; the scale bar is 20 μm. Individual tumor sizes and their volumes are in Table S7.

To evaluate the effects of ponatinib and sirolimus in vivo for tumor growth, 5-week-old athymic nude mice were subcutaneously inoculated with RPMI8226 cells and were treated three times a week with intraperitoneal injection of single or combination ponatinib and sirolimus; they were compared with the PBS injected control mice; treatments commenced when the xenografts became palpable at week 2 post implantation. For treatments, the mice were divided randomly into four groups, and the tumor sizes were measured every three days. The combination treatment of ponatinib and sirolimus was seen to impressively reduce xenograft tumor growth and tumor weight for MM models as compared to tumors for single-agent treatments or PBS control (Figure 6A–C). In addition, no significant increases in serum levels kidney (creatinine and BUN) and liver function enzyme markers alanine transaminase (ALT) and aspartate aminotransferase (AST) were measured in mice treated with the ponatinib (10 mg/kg)/sirolimus (5 mg/kg) combination or vehicle for 14 days (Figure 6D). Similarly, the mice did not display any bodyweight loss from the treatment compounds compared with the control (Figure 6E). The histopathological analysis of tumor samples by hematoxylin-and-eosin (H&E) staining verified that the combination treatment best led to a marked reduction in tumor cellular density, in line with reduced proliferation and cell death (Figure 6F).

### 3.9. Ponatinib/Sirolimus Combination in Xenografts Leads to Best Reduction of HK2 and GPI Activity and Protein and a Corresponding Decrease in Total ATP and Lactate Levels

**Figure 7:**
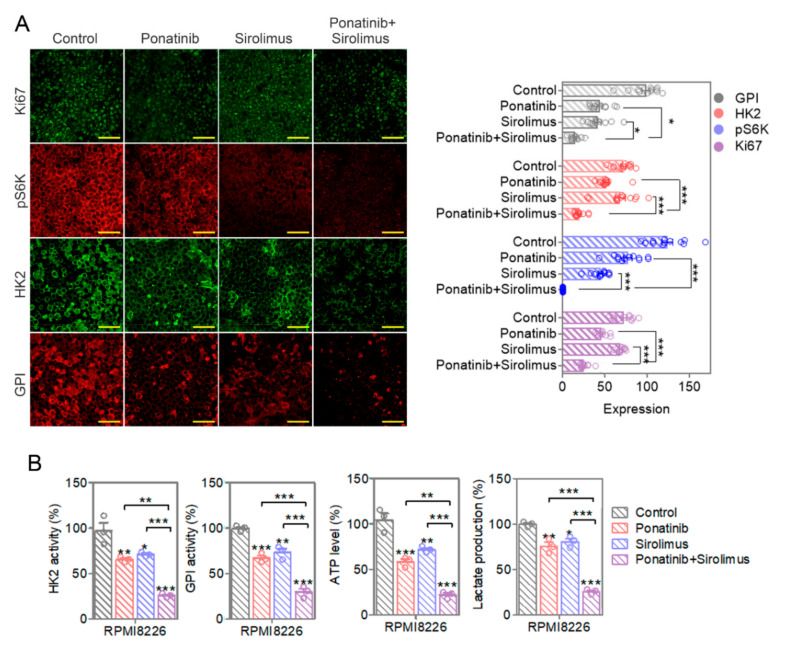
Ponatinib-and-sirolimus combination reduces the levels of Ki67 proliferation marker, mTORC1 activity, and glycolysis rate-limiting enzymes in multiple myeloma tumors. (**A**) Fixed tumor tissue samples were stained with antibodies specific to Ki67 (for proliferation), pS6K (for mTORC1 activity), HK2, and GPI protein levels (for glycolysis). The fluorescent images were quantified by using ImageJ software and plotted as a histogram. Scale bar is 50 μm. (**B**) Tumor tissue lysates were prepared, and HK2 and GPI activity, ATP, and lactate levels were quantified. The data are representative of 3 samples from each group. Data are presented as mean ± SEM; *t*-test: * *p* < 0.05, ** *p* < 0.01, and *** *p* < 0.001.

The immunohistochemistry analysis revealed that the protein levels for the rate-limiting glycolytic enzymes HK2 and GPI were reduced by either ponatinib- or sirolimus-treated RPMI8226 xenografts, but the results were best for the combination treated arm samples (Figure 7A). In addition, the total enzymatic activity for HK2 and GPI in the xenograft samples was also reduced with either ponatinib or sirolimus in the treated xenograft, but most impressively with the combination treatment (Figure 7B). These results are in agreement with the previous observation with the cultured RPMI8226 and U266 cells, with the combination treatment having the best effect in reducing HK2 and GPI levels (Figure 3E and Figure 4A). In agreement with the reduced total activity of the two rate-limiting glycolysis enzymes, the xenograft-associated ATP and lactate levels were similarly reduced in the treatment groups and most robustly in the combination treatment group. This is reflective of the reduced levels of glycolysis as the source of ATP and lactate in the treatment arms.

### 3.10. Enzyme Levels and the Total Activity for HK2 and GPI, along with the Production Levels of ATP and Lactate, Are Reduced in Treatment Arm Xenografts

The levels of Ki67 (encoded by gene *MKI67*) proliferation marker were also reduced in tumor tissues; this was seen most impressively for the combination therapy (Figure 7A). Lactate and ATP levels, along with HK2 and GPI enzymatic activity levels, were also most significantly diminished in the combination therapy with the two drugs (Figure 7B). The relative mRNA level of glycolytic genes showed that combination treatment considerably suppressed HK2 and GPI expression in the xenograft tumor tissues (Figure S3), indicating an in vivo inhibitory effect of ponatinib and sirolimus on tumor metabolism. Taken together, our data indicate that ponatinib is synergistic with sirolimus, supporting the necessity in MM treatment.

### 3.11. Ponatinib-and-Sirolimus Combination Treatment Increases ROS Markers, Protein Disulfide Isomerase (PDI) and C/EBP Homologous Protein (CHOP), in Xenograft Samples

**Figure 8:**
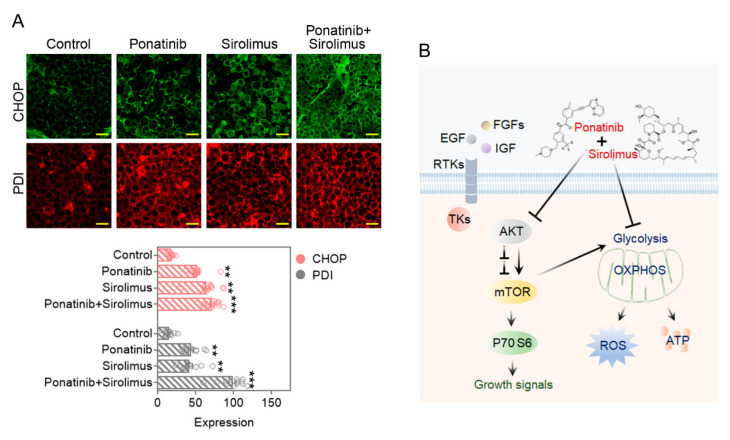
Ponatinib-and-sirolimus combination increases ROS production in multiple myeloma tumors. (**A**) Fixed tumor tissue samples were stained with antibodies specific to CHOP and PDI as ROS markers. The fluorescent images were quantified by using ImageJ software and plotted as a histogram. Scale bar is 50 μm. Data are presented as mean ± SEM; *t*-test: ** *p* < 0.01, *** *p* < 0.001. (**B**) Ponatinib-and-sirolimus combination inhibits glycolysis and OXPHOS to inhibit ATP production and increase ROS accumulation; AKT and mTOR signaling/activity was reduced by the drug combination.

In MM xenograft models, we examined whether the drug treatments also increase the ROS levels. We used the ER stress markers PDI and CHOP as surrogate markers for increased accumulated ROS levels in xenograft samples [28]. Immunohistochemistry analysis data revealed significant increases in the levels of tumor-associated PDI and CHOP, particularly for PDI in the combination drug treated animals (Figure 8A). These findings are similar to the observed increases in ROS levels seen in cultured cells for the various treatments (Figure 5A). From the in vitro experiments, most of the viability loss from single or combination treatment with ponatinib and sirolimus was prevented with the ROS scavenger, NAC, pointing to the combination drug treatment resulting in the buildup of ROS and being the major cause of growth inhibition in these cancers. From our analysis, we noted that the combination of ponatinib and sirolimus in MM cells provides blocks on AKT and mTORC1 (downstream of various growth signals of tyrosine kinases), effectively blocking glycolysis and OXPHOS (both sources of ATP), and also generates ROS levels that prove to be catastrophic for these cells (Figure 8B).

## 4. Discussion

Given the heterogeneity for gene-level mutations that drive various tumor types, the pathways enabling cancer metabolism remain unchanged; these include signals for the upregulation of aerobic glycolysis or the Warburg effect for the majority of cancer types [1]. Given this constancy in elevated glycolysis for cancer cells, it may be thought that targeting the Warburg effect may provide certain selectivity in normal versus cancer for the design of therapeutics. However, this targeting of metabolic pathways for a single gene or by a single pathway inhibitor frequently leads to metabolic reprogramming, allowing escape for the cancer drug away from that block [12,29]. Fortunately, there are only a handful of such reprogramming pathways that the cancer cell can activate, as they all have to provide enough ATP and biomass manufacture for the cell to survive and possibly proliferate at a later time when the therapeutic pressure has lifted [29].

The mTOR pathway, as the nutrient sensor for the cell, regulates various aspects of metabolism, and it has recently been implicated in metabolic reprogramming of model cancer cells that were subjected to a glycolytic block with 2-deoxyglucose (2DG) [12]. 2DG inhibits the enzyme GPI, a rate-limiting enzyme in the glycolysis and relatively early in the pathway [12]. The metabolic escape involved bypassing the initial stages of glycolysis (away from HK2 and GPI) and using the pentose phosphate pathway (PPP) in shuttling the imported glucose in form of glucose 6-phosphate back to the glycolysis pathway. This allows resumption of the entry of the glucose carbons to pyruvate, restarting the production of ATP and biomass. There was also documented increased glutamine consumption to contribute to increased mitochondrial OXPHOS via the TCA cycle. Blocking of the mTORC1 activity with small molecules allowed for negating such metabolic reprogramming (upregulation of OXPHOS and PPP), presumably through blocking of production of sterol regulatory element-binding protein (SREBP), a downstream target of mTORC1 [12].

In this study, we aimed to potentiate the block of glycolysis seen with our lead glycolysis inhibitor, ponatinib, which is an approved cancer drug and a known tyrosine kinase inhibitor [30]. Ponatinib was obtained from our screen of small molecules approved for cancer therapy, from a program run by NCI that allows laboratories to test new indications for approved anticancer drugs. Ponatinib, similar to most small-molecule tyrosine kinase inhibitors, has a relatively broad specificity in blocking tyrosine kinases; it has been documented to block FGFR, BCR-Abl, Abl, c-Kit (encoded by gene *KIT*), and ephrin receptors [18,19,20]. We noticed sizable levels of OXPHOS remaining with in vitro treatment of cells with ponatinib. By combining it with the mTORC1 inhibitor, sirolimus, we observed a synergistic decrease in lactate, pyruvate, and ATP production and block of continued OXPHOS. Significantly, the levels of ATP were potently reduced. The survival signaling pathway for activated AKT was also synergistically inhibited by the combination of the two compounds. Importantly, the levels of destructive ROS were also maximally elevated in treated mice xenografts. We used suboptimal levels of sirolimus and ponatinib to good effect for mouse MM xenograft tumor growth inhibition, and no toxicity was seen in treated mice for kidney/liver function and maintaining weight.

As the sensor of nutrient levels, mTOR provides the means of metabolic reprogramming, as it regulates nutrient uptake and delivery for the cell [12]. From multiple cancer studies, however, mTORC1 inhibitors by themselves have shown limited antitumor efficacy [31]. As discussed above, by combining the mTORC1 with a tyrosine kinase inhibitor ponatinib, additional gains were seen.

The combination of ponatinib and sirolimus generated high levels of ROS both in vitro and for the surrogate markers of ROS in mouse xenografts. The oxidation scavenger NAC was able to recover most of the viability loss from the combination compounds for the cancer cells, pointing to ROS generation as being catastrophic for the MM cells, and being the major factor in contributing to cell death in these cells. In general, cancer cells have higher levels of ROS than normal tissue, and a certain level of ROS is thought to contribute to tumor development by causing DNA mutations and modifying pathway signaling [32]. Given excessive levels of ROS, tumor cells increase their antioxidation activity, but they succumb to ROS stress when not allowed to cope [33]. Inhibition of glycolysis is thought to block the antioxidant pathways in the cell, as pyruvate (by directly scavenging hydroperoxides) and nicotinamide adenine dinucleotide phosphate (NADPH) (by providing the necessary electrons for the thioredoxin and glutathione dependent detoxification enzymes) contribute to ROS scavenging [26,34]. A direct block of mTORC1 also reduces the production of glutathione, a major antioxidant for the cell [32]. The mTORC1 block reduces SREBP levels and diminishes the levels of glucose-6-phosphate dehydrogenase (G6PD) as part of the NADPH production pathway [35]. With a severe block in glycolysis, the ROS levels are no longer under control by the cancer cells, resulting in catastrophic oxidative stress [25].

It should be noted that multiple myeloma frequently develops resistance to inhibition of various pathways [7], although the causes of the developed resistance are frequently not known. There are instances of mTORC1 inhibition leading to rebound activation of MAPK via PI3K/AKT [36,37]. We did not study the rebound MAPK activation, although we did not see increased p-ERK levels with sirolimus (Figure 4A); instead, we observed an additive block on p-ERK levels with the combination of sirolimus/ponatinib (Figure 4A). Ponatinib, as a broad-specific tyrosine kinase inhibitor is known to also block PI3K/AKT and, thus, is thought to be a good combining partner to an mTORC1 inhibitor for overcoming rebound PI3K-dependent MAPK activation [38].

Our study could be followed up by an assay of the twin-drug combination on the tumor microenvironment in an orthotopic (bone-implanted) multiple-myeloma mouse model. A syngeneic mouse model would allow the study of the interaction of the cancer cells with the host immune cells and the environmental bone cells. Although the effect of ponatinib/sirolimus on the bone microenvironment was not the focus of our study, a large interactome analysis of various cell types and their bi-directional messaging would complete the picture of metabolically challenged tumor cells and how they interact with their environment, growing and remodeling the bone [39,40]. It has been documented that the tumor cells re-engineer their bone environment, favoring osteoclast recruitment and activation via interaction of TNFR-related protein receptor activator of NFκB (RANK) (on surface of osteoclasts)-RANK ligand (RANKL) (produced by osteoblasts and T and B cells). Agents that disrupt osteoclastogenesis, such as bisphosphonates and anti-RANKL blocking antibody (denosumab), have shown clinical efficacy in various cancer cases, including multiple myeloma, and could be promising add-ons to the combination cancer-metabolism-targeting agents. An additional therapeutic area is the quality-of-life issue of addressing bone lesions and the generated bone pain in multiple myeloma [41]. A more thorough cause-and-effect analysis is required on the effect of cancer-metabolism targeting therapies on the tumor environment.

## 5. Conclusions

In summary, by combining two approved anticancer drugs, one a tyrosine kinase inhibitor and the other an mTORC1 inhibitor, we obtained synergistic inhibition of tumor growth, thought to occur by causing the onset of catastrophic ROS due to a severe block in glycolysis and residual OXPHOS. Our combination of ponatinib/sirolimus at the doses used also resulted in no manifest toxicity in treated mice for parameters of kidney and liver function or weight loss in treated animals. This study confirms the merit of combining existing drugs for targeting difficult-to-treat tumors.

## Data Availability

The data presented in this study are available in this article and in the Supplementary Materials.

## Supplementary Materials

The following supporting information can be downloaded at https://www.mdpi.com/article/10.3390/cancers14112766/s1. Figure S1: Glycolytic gene expressions are downregulated by 2DG. Figure S2: Ponatinib-and-sirolimus combination treatment. Figure S3: Ponatinib-and-sirolimus combination reduces HK2 and GPI signals in multiple-myeloma tumors. Figure S4: Raw Western blot images for Figure 4A. Figure S5: Raw Western blot images for Figure 4B. Figure S6: Example of the Power*G achieved power calculation. Table S1: List of cancer-patient databases. Table S2: List of primers for qPCR. Table S3: Plate map for Bioneer glucose metabolism PCR array. Table S4: Biochemical assays. Table S5: List of antibodies used for Western blot (WB) and immunohistochemistry (IHC). Table S6: Achieved power calculation from using G*Power version 3.1.9.4. Table S7: Individual tumor dimensions and calculated volume and values of mean, standard deviation (SD), and SEM.
