# Supplementary material for: mTORC1-Inhibition Potentiating Metabolic Block by Tyrosine Kinase Inhibitor Ponatinib in Multiple Myeloma
Source: Cancers (Basel). 2022 Jun 2;14(11):2766. doi: 10.3390/cancers14112766 (PMC9179535; doi:10.3390/cancers14112766)
Supplement: Supplementary file 1 [file cancers-14-02766-s001.zip › cancers-1703078-supplementary.pdf]

cancers

MDPI

Supplementary Materials

mTORC1-Inhibition Potentiating Metabolic Block by Tyrosine Kinase Inhibitor Ponatinib in Multiple Myeloma

Uddin Md. Nazim, Kausik Bishayee, Jieun Kang, Dongkwan Yoo, Sung-Oh Huh * and Ali Sadra *

Department of Pharmacology, College of Medicine and Institute of Natural Medicine, Hallym University, Chuncheon 24252, Korea; nazim@hallym.ac.kr (U.M.N.); kausik@hallym.ac.kr (K.B.); 40442@hallym.ac.kr (J.K.); ydg9611@naver.com (D.Y.)

* Correspondence: s0huh@hallym.ac.kr (S.-O.H.); alisadra@hallym.ac.kr (A.S.)

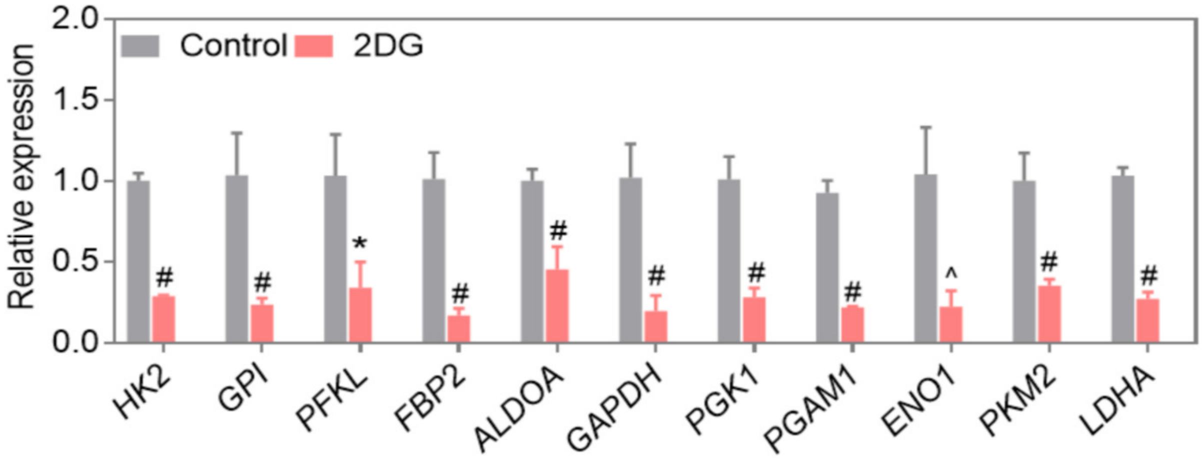

Figure S1. Glycolytic gene expressions are downregulated by 2DG. RPMI8226 cells were treated with 2DG (10 mM) for 12 h; gene-expression changes were examined by qPCR. Data are presented as mean ± SEM; *t*-test: * $p < 0.05$, ^ $p < 0.01$, and # $p < 0.001$.

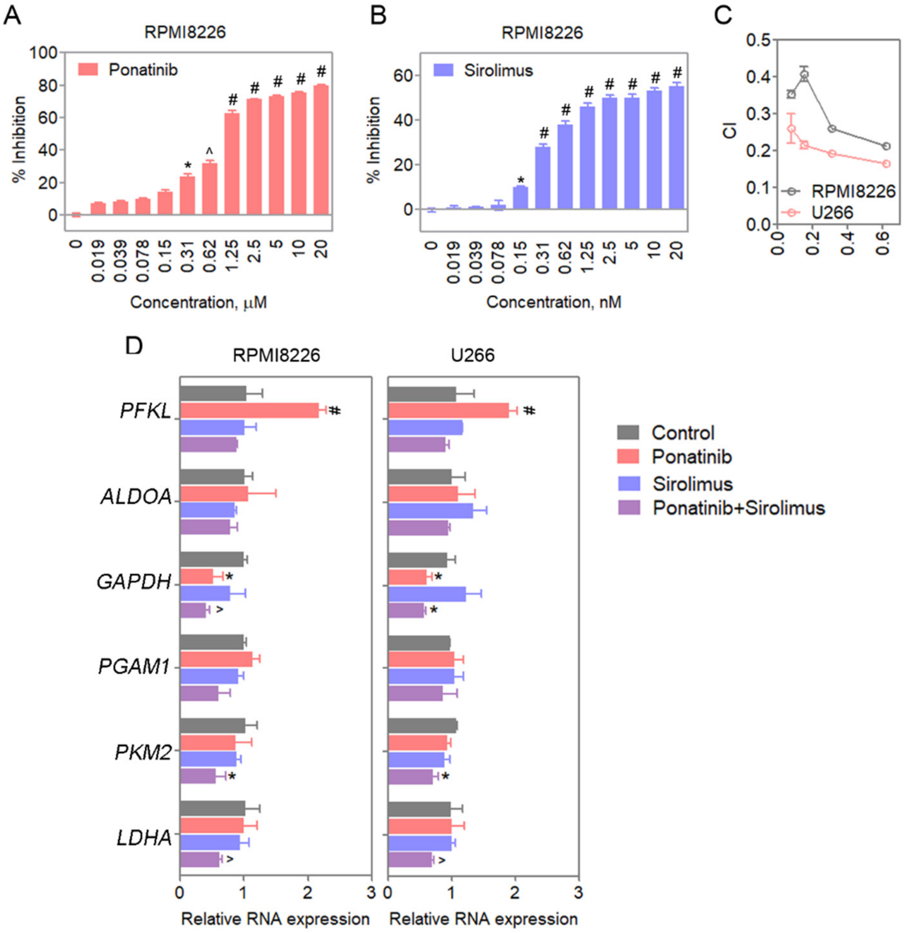

Figure S2. Ponatinib-and-sirolimus combination treatment. (A,B) Cell viability assay was performed on RPMI8226 cells. Cells were incubated with ponatinib or sirolimus at different concentrations for 72 h. (C) CI index was calculated from Figure 3A and plotted by using GraphPad Prism 5 software. (D) RPMI8226 and U266 cells were treated with ponatinib and/or sirolimus for 12 h. Glycolytic gene-expression changes were quantified by qPCR. Data are presented as mean ± SEM; *t*-test: * $p < 0.05$, ^ $p < 0.01$, and # $p < 0.001$.

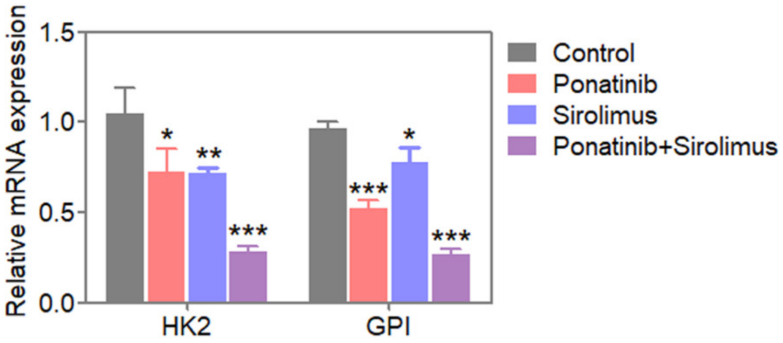

Figure S3. Ponatinib-and-sirolimus combination reduces HK2 and GPI signals in multiple-myeloma tumors. The RNA expression of HK2 and GPI was quantified from the tumor samples by qPCR. Data are presented as mean ± SEM; *t*-test: * $p < 0.05$, ** $p < 0.01$, and *** $p < 0.001$.

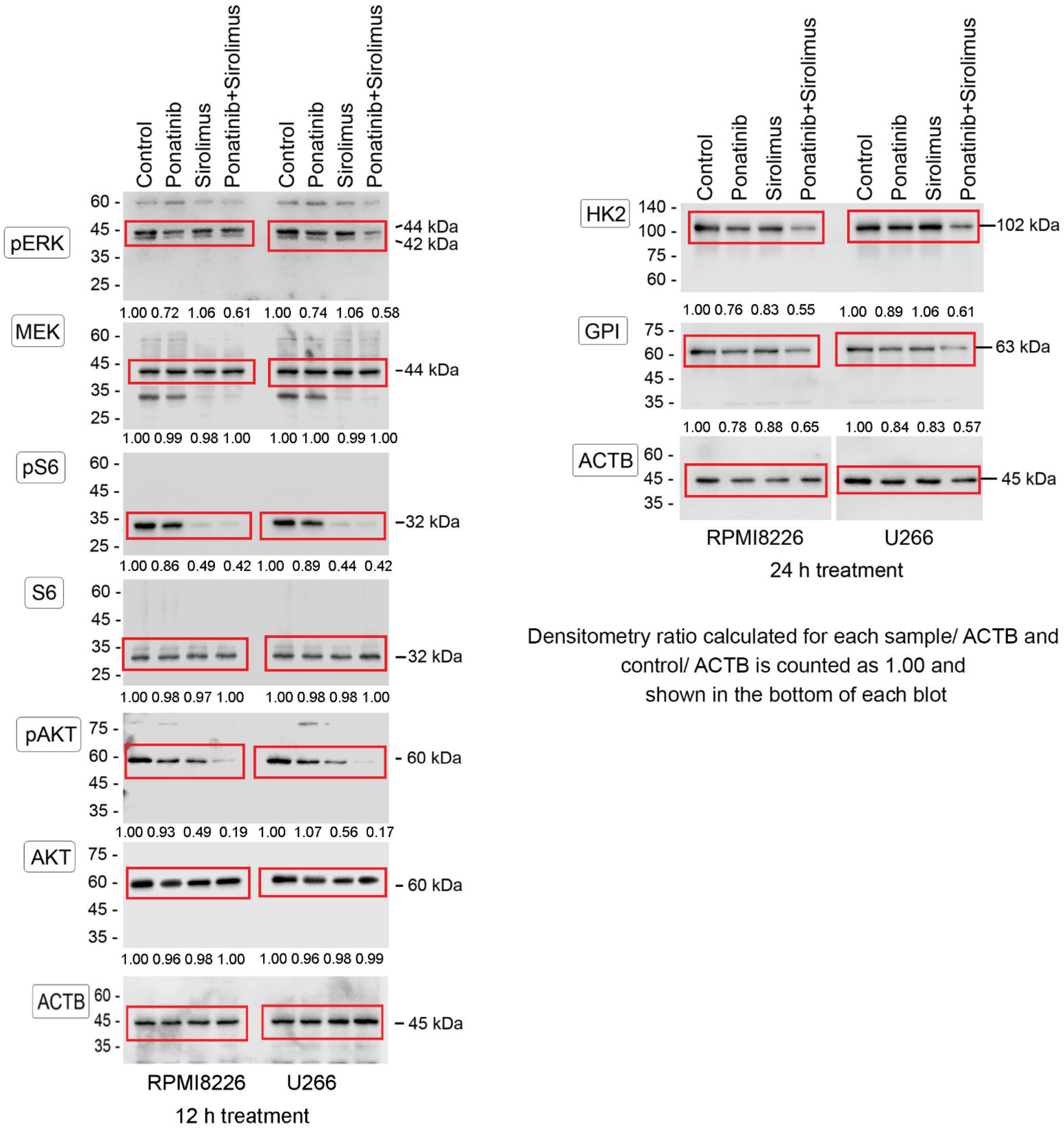

Figure S4. Raw Western blot images for Figure 4A.

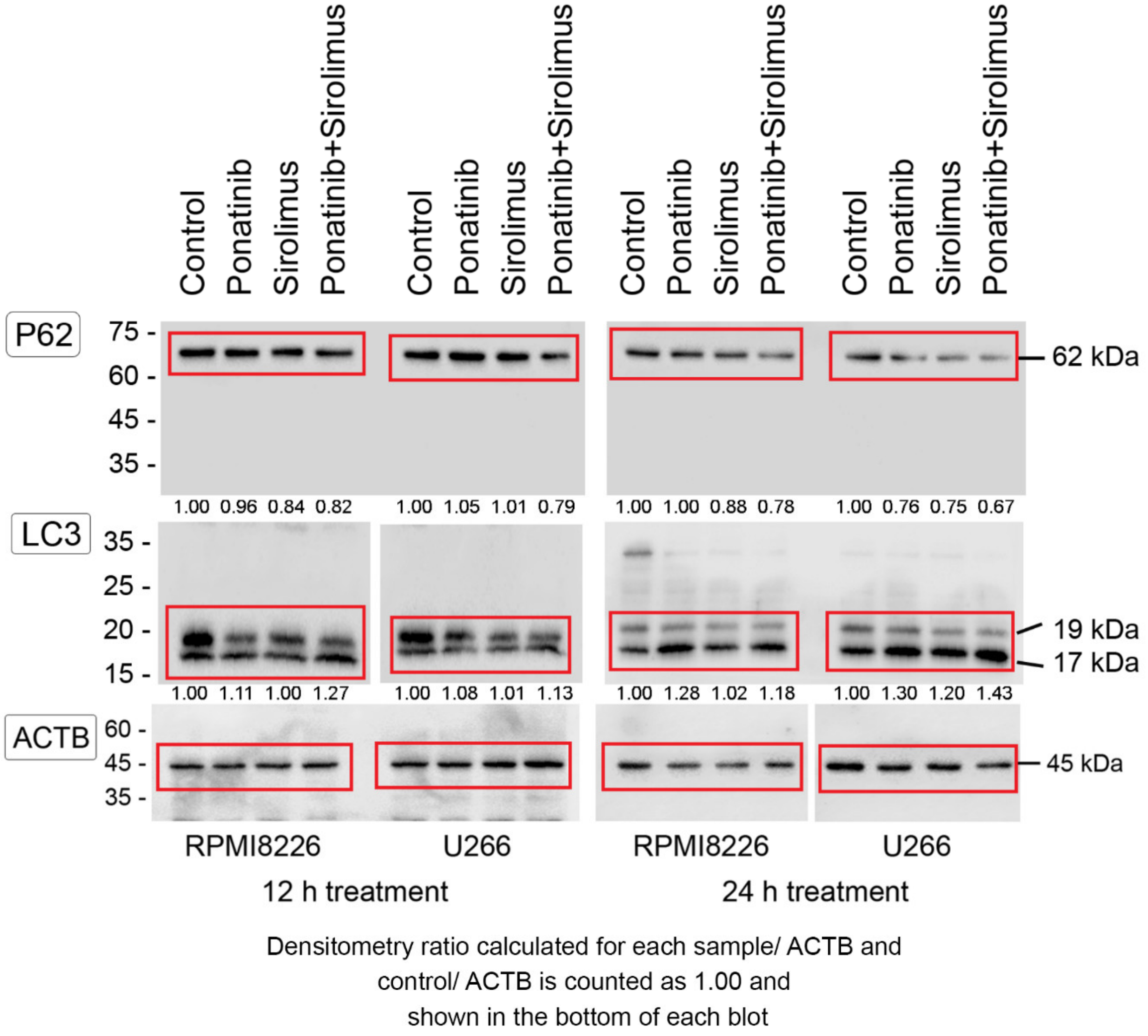

Figure S5. Raw Western blot images for Figure 4B.

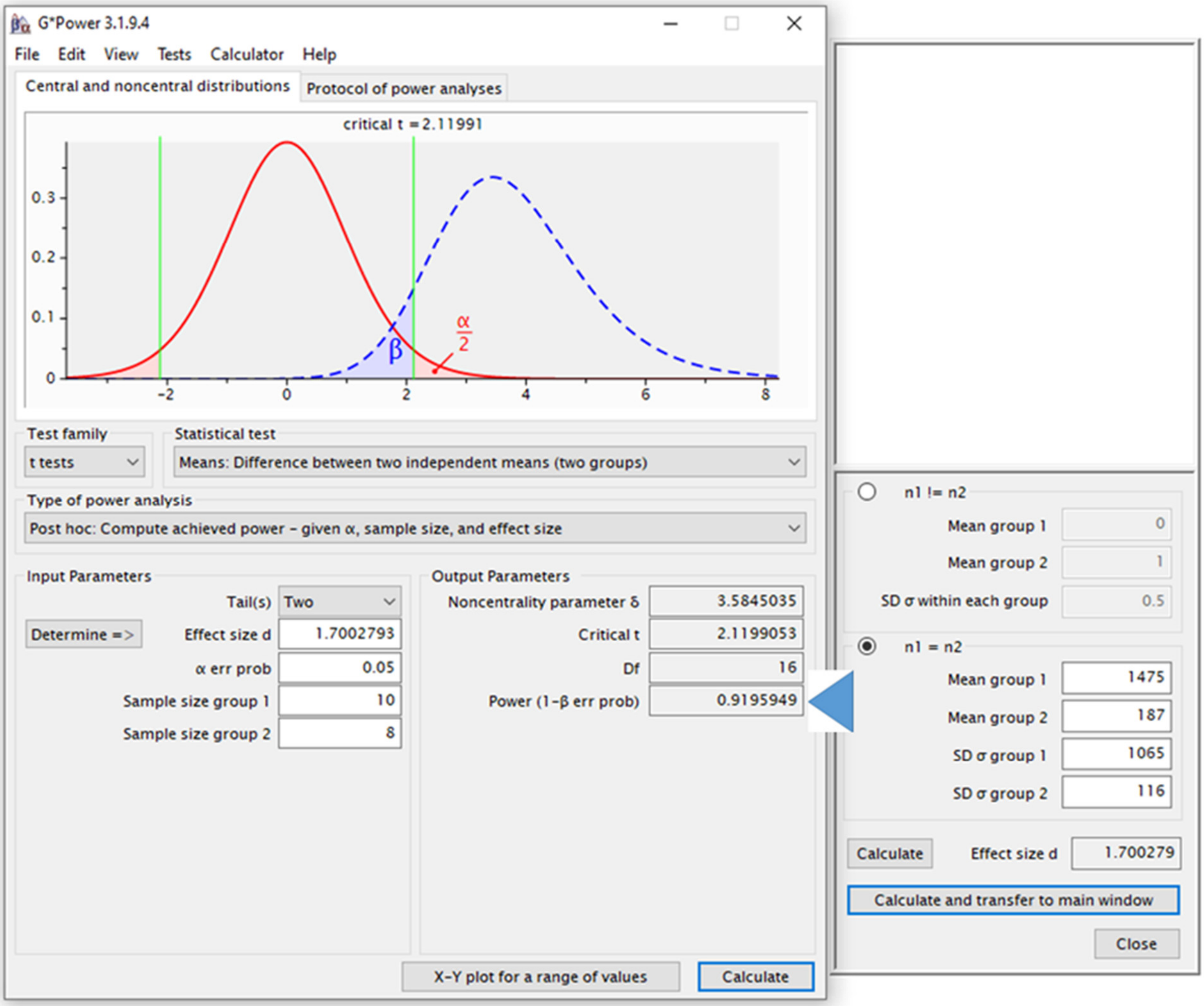

EXAMPLE (see Table for the all the comparisons):

- 2-sample t-test (also known as the independent t-test or Student t-test) (2-tail) (CONTROL vs. DRUG COMBO)
- Post hoc: Compute achieved power – given α, sample size, and effect size
- Effect size d = unknown; use G*Power calculator based on the experimental data set (CONTROL vs. DRUG)
- α=0.05; Power= 1-β; Sample size (given)

Figure S6. Example of the Power*G achieved power calculation.

Table S1. List of cancer-patient databases.

No.	Cancer type	Database	ID	Number of patients
1	Multiple myeloma	Hanamura	GSE2658	542
2	Normal B cells	JIMA	GSE12366	12

Table S2. List of primers for qPCR.

No.	Gene	Vendor	Primer sequence
1	*HK2*	Bioneer	F-CACAGAATTTGATGTGGCTG;<br>R-ATGTTACGGACAATCTCACC
2	*GPI*	Bioneer	F-CTCTATTGTGTTCACCAAGC;<br>R-TGTTGATGTCCCAGATGATG
3	*PFKL*	Bioneer	F-GGAGAAGCTGCGCGAGGTTTAC;<br>R-ATTGTGCCAGCATCTTCAGCATGAG
4	*ALDOA*	Bioneer	F-GAGGAGTATGTCAAGCGAG;<br>R-TAATAGGCGTGGTTAGAGAC
5	*GAPDH*	Bioneer	F-TTCCGTGTCCCCACTGCCAACGT;<br>R-CAAAGGTGGAGGAGTGGGTGTCGC
6	*PGK1*	Bioneer	F-ATGCTGAGGCTGTCACTCGG;<br>R-CACAGCAAGTGGCAGTGTCTCC
7	*PGAM1*	Bioneer	F-ATTGTCAAGCATCTGGAGG;<br>R-TAGGCTTCAAGTTCTTGTCC
8	*ENO1*	Bioneer	F-GACTTGGCTGGCAACTCTG;<br>R-GGTCATCGGGAGACTTGAA
9	*PKM2*	Bioneer	F-TGATGGGCTTATTTCTCTCC;<br>R-AAGGTTCACACCCTTCTTG
10	*LDHA*	Bioneer	F-GGAGTTCACCCATTAAGCTG;<br>R-TCAGAGAGACACCAGCAAC
11	*ACTB*	Bioneer	F-CAAGATCATTGCTCCTCCTG;<br>R-GTCATACTCCTGCTTGCTG

Table S3. Plate map for Bioneer glucose metabolism PCR array (Catalog number SH-0000-10).

	1	2	3	4	5	6	7	8	9	10	11	12
A	*PRPS1*	*AGL*	*GCK*	*PGM1*	*PCK1*	*PDK4*	*IDH1*	*RBKS*	*PGM2*	*FBP2*	*PCK2*	*PHKB*
B	*PGK1*	*GYS2*	*TKT*	*PC*	*PKLR*	*PRPS2*	*DLD*	*MDH1B*	*ALDOC*	*PGK2*	*GPI*	*RPIA*
C	*PHKG1*	*TPI1*	*SDHD*	*G6PC3*	*ENO2*	*PGM3*	*PDK3*	*IDH3G*	*UGP2*	*GBE1*	*SUCLG1*	*GALM*
D	*G6PC*	*H6PD*	*PYGM*	*PDPR*	*TALDO1*	*G6PD*	*SDHB*	*PFKL*	*SDHC*	*IDH2*	*PDHA1*	*IDH3B*
E	*SUCLG2*	*PDK1*	*ENO3*	*DLAT*	*MDH2*	*ENO1*	*GSK3B*	*PGLS*	*MDH1*	*PYGL*	*PGAM2*	*PHKG2*
F	*CS*	*ACLY*	*DLST*	*PHKA1*	*GSK3A*	*ALDOB*	*ALDOA*	*PDP2*	*FH*	*PRPS1L1*	*SUCLA2*	*SDHA*
G	*ACO2*	*RPE*	*FBP1*	*HK2*	*GYS1*	*HK3*	*BPGM*	*PDHB*	*OGDH*	*ACO1*	*PDK2*	*IDH3A*
H	*ACTB*	*B2M*	*GAPDH*	*GUSB*	*HPRT1*	*PPIA*	*RPL13A*	*RPLP0*				

Table S4. Biochemical assays.

No.	Assay	Vendor	Catalog Number
1	OXPHOS	Cayman Chemical (Ann Arbor, MI, USA)	600800
2	Lactate production assay	Abcam (Cambridge, UK)	ab65331
3.	Pyruvate production assay	SOLARBIO (Beijing, China)	BC2205
4.	ATP production assay	Biomax (Seoul, South Korea)	BM-ATP100
5.	Glucose uptake assay	Abcam	ab136955
6.	HK2 activity assay	Abcam	ab136957
7.	GPI activity assay	BioVision (Milpitas, CA, USA)	K775-100

Table S5. List of antibodies used for Western blot (WB) and immunohistochemistry (IHC).

No.	Antibody	Species	Applications	Vendor	Catalog Number
1	LC3A/B	Rabbit	WB	Cell Signaling Technology	12741S
2	HK2	Mouse	IHC, IHC	Abcam	ab104836
3	GPI/AMF	Mouse	IHC, IHC	Abcam	ab66340
4	P62	Rabbit	WB	Cell Signaling Technology	14354S
5	pS6 (Ser240/244)	Rabbit	WB, IHC	Cell Signaling Technology	5364
6	pERK (Thr202/Tyr204)	Rabbit	WB	Cell Signaling Technology	9101L
7	MEK	Mouse	WB	Cell Signaling Technology	4694
8	S6	Mouse	WB	Cell Signaling Technology	2317S
9	Actin, beta	Rabbit	WB	Cell Signaling Technology	4967
10	pAKT (Ser473)	Rabbit	WB	Cell Signaling Technology	4060
11	AKT	Rabbit	WB	Cell Signaling Technology	9272
12	Ki67	Rabbit	IHC	Abcam	ab15580
13	CHOP	Mouse	IHC	Cell Signaling Technology	2895S
14	PDI	Rabbit	IHC	Cell Signaling Technology	3501S

Table S6. Achieved power calculation from using G*Power version 3.1.9.4.

Means Difference Test of Two Independent Groups/t-test/2-Tail	α	Calculated Effect Size (d)	Sample Size	Mean Group 1 (mm^3)	SD Group 1 (mm^3)	Mean Group 2 (mm^3)	SD Group 2 (mm^3)	Achieved Power
Control vs. combination drug (step-by-step calculation shown below)	0.05	1.7002793	10 for control and 8 for drug combo (we started with 10, but 2 could not be measured/harvested, as there was no tumor left at the end of the study)	1475	1065	187	116	0.9195949 (example shown in Figure S6)
Control vs. ponatinib	0.05	0.8683838	10 each	1475	1065	779	388	0.4515322
Control vs. sirolimus	0.05	1.1262898	10 each	1475	1065	612	200	0.6637054
Ponatinib vs. sirolimus	0.05	0.5410456	10 for single drug and 8 for drug combo (explained above)	779	388	612	200	0.2087642
Combination drug vs. ponatinib	0.05	2.1581496	10 for single drug and 8 for drug combo (explained above)	161	116	779	388	0.9893559
Combination drug vs. sirolimus	0.05	2.7586291	10 each	161	116	612	200	0.9997499

Table S7. Individual tumor dimensions and calculated volume and values of mean, standard deviation (SD), and SEM.

		H	W		H	W		H	W		H	W		
			1	V (mm3)	2	2	V (mm3)	3	3	V (mm3)	4	4	V (mm3)	
CONTROL	1	9	6.6	196.02	10.7	8.7	404.9415	16.5	14.1	1640.183	19.4	17.5	2970.625	
	2	7.1	6.7	159.3595	10.5	8	336	13.2	12	950.4	20.4	17.9	3268.182	
	3	6	4.3	55.47	10.8	7.8	328.536	19.5	14.5	2049.938	12.7	12.1	929.7035	
	4	5.6	5.5	84.7	13.2	8.7	499.554	10.5	8.5	379.3125	9.8	9.6	451.584	
	5	7.3	6.1	135.8165	13.4	9.4	592.012	9.8	7.9	305.809	17.8	7.5	500.625	
	6	6.7	4.5	67.8375	9.2	6.7	206.494	14	13.2	1219.68	17.7	16.1	2294.009	
	7	6.4	4.3	59.168	11.3	9.6	520.704	15.6	14	1528.8	16.5	14.5	1734.563	
	8	7.4	6.1	137.677	8.9	6.2	171.058	6.5	4.5	65.8125	12.2	10.2	634.644	
	9	5	3.4	28.9	11.3	10.4	611.104	12.5	10.5	689.0625	14.7	14.3	1503.002	
	10	3.2	2.1	7.056	12	8.4	423.36	11.6	9.2	490.912	12.3	8.7	465.4935	
													1475.243	MEAN
													336.8277	SEM
													1065.143	SD
PONATINIB	1	4.6	4.5	46.575	10.2	6.4	208.896	13	10.7	744.185	14.7	13.6	1359.456	
	2	8.4	5.4	122.472	4.5	4.5	45.5625	13.1	9	530.55	13.1	11.3	836.3695	
	3	5.7	4.4	55.176	6	4.5	60.75	12.7	10.4	686.816	11.8	9.9	578.259	
	4	9.3	4.5	94.1625	9.9	8.4	349.272	7.4	8.6	273.652	15.5	6.5	327.4375	
	5	5.3	3.8	38.266	6.4	5.3	89.888	10.9	8.9	431.6945	8.5	6	153	
	6	6.4	3.5	39.2	6.8	5.9	118.354	6	5.1	78.03	14.3	10.1	729.3715	
	7	8.9	4.5	90.1125	11.5	10.3	610.0175	11	10.6	617.98	12.7	11.8	884.174	
	8	8.4	4.8	96.768	9.8	8.9	388.129	11.6	8.7	439.002	13	12	936	
	9	9	8.2	302.58	10.4	6.5	219.7	10.4	5.7	168.948	16.7	12.7	1346.772	
	10	7.9	5.6	123.872	15.5	6	279	10.6	7.7	314.237	13.4	9.8	643.468	
													779.4307	MEAN
													122.7105	SEM
													388.0446	SD
SIROLIMUS	1	6.8	6.3	134.946	11.2	7.8	340.704	8.5	7.8	258.57	14.5	8.7	548.7525	
	2	4.5	3.4	26.01	5.9	4.4	57.112	10.3	8.4	363.384	17.2	10.4	930.176	
	3	8	2.9	33.64	10.4	6.8	240.448	10.8	7.5	303.75	11	9.8	528.22	
	4	5	4.5	50.625	11.9	5.5	179.9875	10.8	8	345.6	13.4	11	810.7	
	5	4.1	3.5	25.1125	8.5	6	153	9.5	7.9	296.4475	10.6	8.4	373.968	
	6	5.1	4.5	51.6375	13.3	9	538.65	11.7	8.9	463.3785	10.9	9	441.45	
	7	5.9	4	47.2	8.3	7	203.35	4.5	4.5	45.5625	12.2	10.2	634.644	
	8	5.9	4.2	52.038	11.3	8.8	437.536	10.4	9.7	489.268	12.5	12	900	
	9	8.2	4.6	86.756	7.9	6.7	177.3155	6.9	5.8	116.058	11	9	445.5	
	10	3.4	2.1	7.497	9	7.2	233.28	6.2	4.4	60.016	11.5	9.4	508.07	
													612.1481	MEAN
													63.19843	SEM
													199.851	SD
DRUG COMBINATION	1	4.6	3.8	33.212	5.4	4.9	64.827	4.3	4.2	37.926	8	7	196	
	2	8.4	6.5	177.45	7.1	6.5	149.9875	7.3	5.8	122.786	9.4	8.9	372.287	
	3	7.1	5.5	107.3875	7.2	5.1	93.636	6	4.2	52.92	9	4.6	95.22	
	4	8	6.1	148.84	9.2	6.2	176.824	8.8	5.2	118.976	6	5.4	87.48	
	5	5.6	5.1	72.828	9.2	7.6	265.696	7.9	5.6	123.872	8	6.1	148.84	
	6	6.6	5	82.5	8.4	6.8	194.208	8	5.6	125.44	8.2	5.5	124.025	
	7	7.3	5.6	114.464	7.3	6.1	135.8165	4.5	4.2	39.69	10	8.5	361.25	
	8	4.5	4.5	45.5625	8.3	6.6	180.774	4.5	3.2	23.04	6.9	5.6	108.192	
	9 *	5.7	4.7	62.9565	11	7.2	285.12	6.1	5.4	88.938	4.5	4.5	45.5625	
	10 *	7.2	5	90	10.5	8.3	361.6725	9.8	5.7	159.201	6.3	4.9	75.6315	
													186.6618	MEAN
													41.13023	SEM
													116.3339	SD

* 2 tumors excluded; they were so tiny that they could not be harvested at the end of the study and accurately weighed/pictured
